# An integrated analysis of the effects of microRNA and mRNA on esophageal squamous cell carcinoma

**DOI:** 10.3892/mmr.2015.3557

**Published:** 2015-03-27

**Authors:** YONG YANG, DIANBO LI, YANG YANG, GENING JIANG

**Affiliations:** 1 Department of Thoracic Surgery, Shanghai Pulmonary Hospital Affiliated Tongji University, Shanghai 200433, P.R. China; 2 Department of Thoracic Surgery, Linyi Tumor Hospital, Linyi, Shandong 276001, P.R. China

**Keywords:** esophageal squamous cell cancer, microRNA expression, mRNA expression, pathway analysis, regulation network

## Abstract

Esophageal squamous cell cancer (ESCC) is an aggressive type of cancer with poor prognosis and leading to decreased quality of life. The identification of patients at increased risk of esophageal squamous cell cancer may improve current understanding of the role of micro (mi)RNA in tumorigenesis, since the miRNA pattern of these patients may be associated with tumorigenesis. In the present study, the miRNA and mRNA expression profiles of ESCC tissue samples and adjacent normal control tissue samples were obtained from two dependent GEO series. Bioinformatics analyses, including the use of the Gene Oncology and Kyoto Encyclopedia of Genes and Genomes databases, were used to identify genes and pathways, which were specifically associated with miRNA-associated ESCC oncology. A total of 17 miRNAs and 1,670 probes were differentially expressed in the two groups, and the differentially expressed miRNA and target interactions were analyzed. The mRNA of miRNA target genes were found to be involve 49 GO terms and 14 pathways. Of the genes differentially expressed between the two groups, miRNA-181a, miRNA-202, miRNA-155, FNDC3B, BNC2 and MBD2 were the most significantly altered and may be important in the regulatory network. In the present study, a novel pattern of differential miRNA-target expression was constructed, which with further investigation, may provide novel targets for diagnosing and understanding the mechanism of ESCC.

## Introduction

Esophageal cancer is the sixth most life-threatening type of cancer worldwide (1). Unlike the epidemiologic feature of esophageal cancer in the west, in 2005, >90% of the cases of esophageal cancer are esophageal squamous cell carcinoma (ESCC) in China and Japan (2). Despite advances in the diagnosis and treatment of cancer, improvements in esophageal cancer have progressed more slowly and the overall prognosis for patients with ESCC has remained unchanged. Furthermore, variability in the clinical course of patients with ESCC remains to be elucidated, and conventional clinicopathological parameters fail to assist in this. The identification of novel prognostic factors may enable rational selection of the most appropriate therapeutic options for individual patients. Transcriptional profiling using DNA microarray analysis is proving to be a useful tool in cancer research. It has provided novel treatment targets and prediction models for prognosis and treatment response (3–5). An improved understanding of the genetic and molecular mechanisms underlying the disease is key to enabling the early diagnosis, appropriate treatment and improved prognosis of patients with ESCC.

Micro (mi)RNAs are a class of small, non-coding RNAs, which are 20–24 nucleotides in length and function as regulators of gene expression. Each miRNA is considered to be involved in the post-transcriptional regulation of hundreds of genes, and the translational inhibition of a given gene may require binding of more than one miRNA (6). It is estimated that one third of the genes in the human genome are regulated by miRNAs (7) and, to date, >1,800 miRNA genes have been identified (miRBase release 20.0; http://www.mirbase.org/) (8), including several that are involved in key cellular processes, including apoptosis, proliferation and differentiation. (9). MiRNA misexpression or mutation results in a gain or loss of miRNA function and, therefore, a downregulation or upregulation of the target protein. Notably, the successful use of antagomirs to silence miRNAs in mice (10) and in non-human primates (11) suggests the possible therapeutic use of miRNAs. Previously, miRNAs have also been identified as oncogenes or tumor suppressors (12,13).

However, the regulation of miRNAs and corresponding target mRNAs during the occurrence and development of ESCC have not been reported. The advent of genome-wide technologies, including gene expression microarrays, has made it possible to achieve a comprehensive view of the miRNAs and mRNAs alteration involved in ESCC, and the use of bioinformatics enables analysis of differences between miRNAs and mRNAs.

To identify the miRNAs and mRNAs that are involved in the molecular biological changes of ESCC, the present study examined the gene expression microarray of miRNAs and mRNAs from a published database to discriminate those involved in ESCC from those in normal tissues. The results may assist in identifying novel targets for ESCC therapy and provide biomarkers on diagnosis and prognosis.

## Materials and methods

### Selection of patients data

Patient microarray data were obtained from an miRNA and an mRNA datasets, which included 88 and 358 appropriate samples, respectively. The miRNA microarray series contained 44 ESCC tumor and 44 normal control samples, and the mRNA microarray series contained 179 ESCC tumor and 179 normal control samples. The two series were accessible at the NCBI GEO database (http://www.ncbi.nlm.nih.gov/geo/), and their accession numbers were GSE13937 and GSE53625, respectively. The details of sample characteristics were presented in their original articles (14,15).

### Differentially expressed miRNAs

The miRNAs, which were differentially expressed between the ESCC and normal control samples were identified using the limma method, which is a linear model for microarray data analysis (16). The threshold values were set at P<0.05 and false discover rate (FDR)<0.05, from which the ESCC-associated differentially expressed miRNAs were identified.

### Differentially expressed mRNAs

The mRNAs, which were differentially expressed between the ESCC and normal control samples were also identified using the limma method. The P-value and the fold change were calculated for each differentially expressed gene. The thresholds were set at: Fold change>3, P<0.001 and FDR<0.001, from which the ESCC-associated differential expression genes were selected. Unsupervised hierarchical clustering was performed with Cluster (version 3.0; Eisen Lab, Stanford, CA, USA) using Pearson’s correlation distance metric and average linkage, followed by visualization using Treeview (Eisen Lab, Stanford, CA, USA) (17).

### Gene Ontology (GO) analysis

Based on the GO Database (http://www.geneontology.org/), the significant GO terms of the ESCC-associated differentially expressed genes were analyzed with a two-tailed Fisher’s exact test and χ^2^ test using the Database for Annotation, Visualization and Integrated Discovery (http://david.abcc.ncifcrf.gov/home.jsp) analysis (18). The differentially expressed genes were analyzed independently, according to the upregulation and down-regulation of these genes. The P-values of each differentially expressed gene in all the GO terms were calculated. P<0.05 was considered to indicate a statistically significant difference.

### Pathway analysis

Pathway analysis was used to determine the significant pathway of the differential genes, according to the KEGG (http://www.genome.jp/kegg/), Biocarta (http://www.biocarta.com/) and Reatome (http://www.reactome.org/) pathway databases. Fisher’s exact test and a χ^2^ test were used to select the significant pathway, and the threshold of significance was defined by the P-value and FDR (19-21).

### Annotation of miRNA targets

The target mRNAs of the miRNAs were predicted based on TargetScan (http://www.targetscan.org/) version 6.2. TargetScan predicts the biological targets of miRNAs by identifying conserved 8mer and 7mer sites, which match the seed region of each miRNA (7). It also identifies sites with mismatches in the seed region that are compensated by conserved 3′ pairing (22). In mammals, the predictions are ranked based on the predicted efficacy of targeting, calculated using the context scores of the site alignments (23,24). TargetScan Human (http://www.targetscan.org/) considers matches to annotate human untranslated regions and their orthologs, defined by UCSC whole-genome alignments (25). Conserved targeting is also detected within open reading frames.

### miRNA-gene network

The associations between the miRNAs and genes were determined by their counting their differential expression values, and according to the interactions of miRNAs and genes in the Sanger miRNA database (http://www.sanger.ac.uk/) to construct the miRNA-gene network. The adjacency matrix of microRNA and genes, A = [a_i,j_], was obtained from the attribute associations among the genes and miRNAs, where a_i,j_ represents the association weight between the gene (i) and miRNA (j). In the miRNA-gene network, a circular node represents the gene and a square node represents the miRNA, and their association is represented by a line. The center of the network is presented as the degree, which indicates the contribution of one miRNA to the surrounding genes, or the contribution of one gene to the surrounding miRNAs. The key miRNA and gene in the network always have the largest degrees.

### Data analysis

Numerical data are presented as the mean ± standard deviation. Differences between the means were analyzed using Student’s t-test. All statistical analyses were performed using SPSS 13.0 software (SPSS, Inc., Chicago, IL, USA).

## Results

### Clinical characteristics of the two group samples

The same clinical characteristics of the miRNA microarray and mRNA microarray groups were collected from the original articles (Table I). On comparing the two groups, the patients from the miRNA group had similar levels of alcohol and tobacco consumption to those in the mRNA group. In the miRNA microarray group, the distribution of samples in the four tumor-node-metastasis (TNM) stages were similar, while the samples from the mRNA microarray samples were in stages I-III. However, these difference were not statistically significant.

### Overview of the miRNAs profiles

From the miRNAs expression profiles, differentially expressed miRNAs were identified between the ESCC and normal control samples. The miRNA expression profiles were determined by calculating the log fold change in the ESCC group / normal group. Due to a limited sample size, the FDR and P-values were considered, obtaining 17 results. Compared with the normal tissues, the FDR values of miR-375, miR-26a-1^*^ and miR-378 were the most significantly upregulated miRNAs, while miR-21, miR-146b-5p and miR-155 were the most significantly downregulated (Table II). The number of upregulated miRNAs was similar with the number of downregulated miRNAs in the ESCC group.

### Overview of the mRNAs profiles

**Figure 1:**
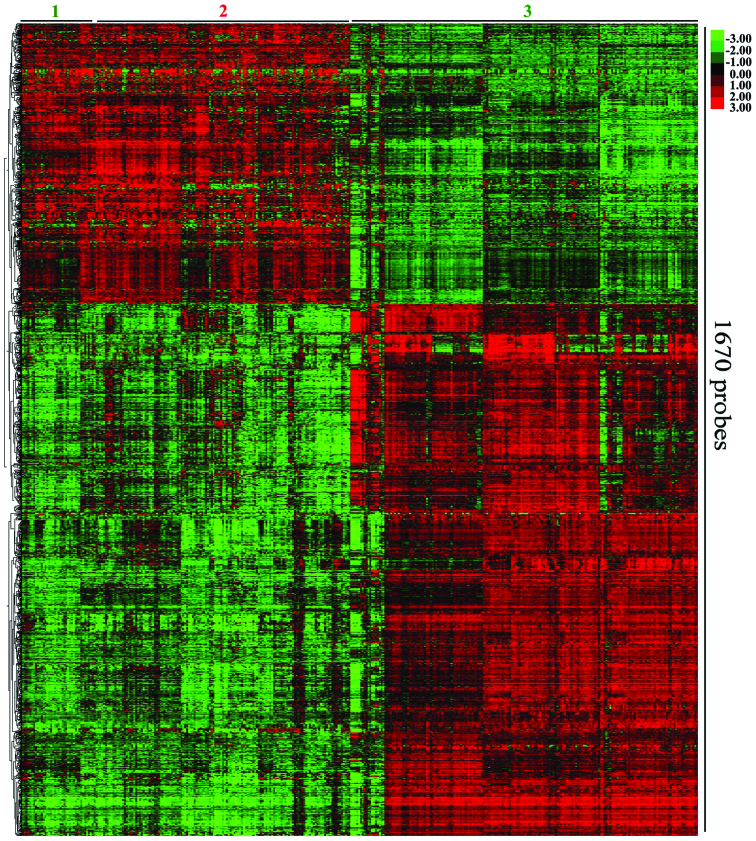
Unsupervised classification of ESCC samples and normal control samples based on mRNA expression profiling. The mRNA expression data are depicted as a data matrix, with each row representing a probe and each column representing a sample. Expression levels are depicted according to the color scale, shown at the top. Red and green indicate expression levels, above and below the median, respectively. The magnitude of deviation from the median is represented by the color saturation.

In the mRNA microarray group, up to 26,154 coding transcripts were detected in the 358 samples. Using the limma method, with cut of criteria of a fold change>3, and P-value and FDR<0.001 between the two groups, 576 probes were upregulated and 1,094 probes were downregulated in the ESCC samples. The global mRNA expression patterns were then evaluated by hierarchical clustering. The most variably expressed mRNAs revealed two major clusters, which correlated with the differentiation state of the tumor (Fig. 1). Expression cluster 2 contained all the ESCC samples, while the normal control groups were divided into sub clusters 1 and 3. COL1A1 was the most significantly upregulated mRNA, and EMP1 was the most significantly downregulated mRNA (Table III). In the ESCC group, a higher number of downregulated mRNAs were observed compared with upregulated mRNAs.

### Microarray-based GO analysis

The target mRNAs for differentially expressed miRNAs were predicted using TargetScan (http://www.targetscan.org/), which revealed 5,532 associations between the two. The intersection set for the predicted target mRNAs and differentially expressed mRNAs from GSE53625, mentioned above, was selected. Following negative correlation, the eligible mRNAs were then used for GO analysis. The threshold of GO terms, which were significantly regulated by miRNAs was P<0.05. The GO terms with the highest levels of enrichment, targeted by miRNAs, included collagen fibril organization and phosphate metabolic process (Tables IV and V).

### Microarray-based pathway analysis

**Figure 2:**
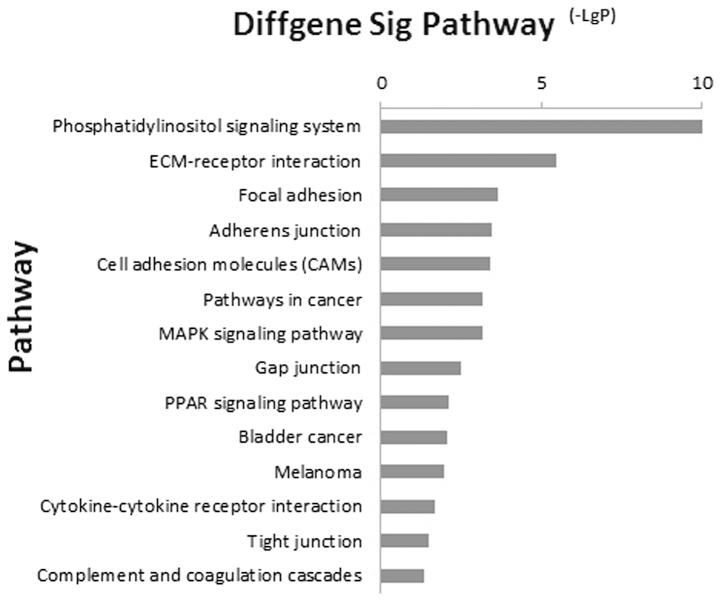
Histogram of signaling pathways, which different significantly between the ESCC and normal samples. X-axis, negative logarithm of the P-value (-LgP); Y-axis, pathway. The higher the -LgP, the lower the P-value. ECM, extracellular matrix; MAPK, mitogen-activated protein kinase; PPAR, peroxisome proliferator-activated receptor.

As signal transduction may be involved in ESCC, the associated pathways were analyzed, according to the functions and interactions of the differential genes. By using Pathway analysis, which considered the relative change direction and fold change and had a the threshold of significance of P<0.05, 14 significant pathways were found (Fig. 2). The most enriched pathways targeted by dysregulated mRNAs included the phosphatidylinositol signaling system, ECM-receptor interaction and focal adhesion pathways and the peroxisome proliferator activated receptor signaling pathway. This suggested that miRNA regulated oncogenesis of ESCC through these pathways.

### miRNA-mRNA network

**Figure 3:**
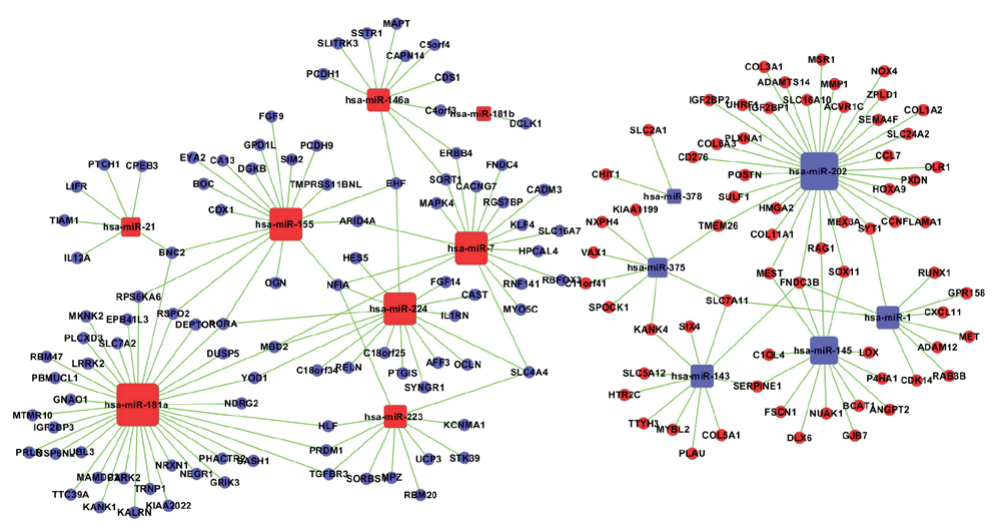
miRNA-mRNA interaction network of esophageal squamous cell carcinoma. Circular nodes represent mRNAs and square nodes represent miRNAs. Blue represents downregulation and red represents upregulation. Solid lines indicate regulatory associations between the miRNAs and mRNAs.

As the pathways identified did not appear to be closely relevant to ESCC, the intersection set of significantly differentially expressed mRNAs, identified by GO and pathway analysis, were screened out. The miRNA-mRNA regulatory networks based on these mRNAs (Fig. 3), distinguished the putative target mRNAs between the overexpressed and underexpressed miRNAs. The total numbers of mRNAs and miRNAs in the network were 164 and 14, respectively. The particular associations between them are listed in Table IV. In the network, the circular nodes represent mRNAs, square nodes represent miRNAs, and lines between two nodes represent interactions between the miRNA and mRNA. The degree represents the number of target genes regulated by particular miRNAs, and the higher the degree, the more central the miRNAs is within the network. miR-181a, miR-202 and miR-155 were identified as the three dysregulated miRNAs with the most target mRNAs, whereas FNDC3B, NFIA and BNC2 were targeted by the most miRNAs.

### Effects of miRNAs on patients

As miRNAs may be important in regulating the formation and development of ESCC from different aspects, the present study examined the core miRNAs in the miRNA-mRNA network of the tumor sample group alone. Clinical characteristics, including alcohol consumption, smoking status, TNM staging and nodal involvement were included in the analysis. miR-181a was expressed more markedly in the late stage (Stage III and IV; P=0.01), while smoking patients were more likely to exhibit overexpression of miR-155 (P=0.01). Notably, no significant differences were observed between the core downregulated miRNAs, including miR-202, miR-145 and miR-143 and the above-mentioned clinical characteristics.

## Discussion

Understanding the clinical relevance of miRNA expression patterns in ESCCs is a necessary to better classify these heterogeneous types of tumor and to circumvent the therapeutic challenges faced upon their clinical management. However, for miRNAs indirectly regulating the pathophysiological process of ESCC, the possible target mRNAs remain to be fully elucidated. The difficulties in using miRNA micro-arrays to predict patients with ESCC arise predominantly due to the challenge in interpreting the numerous complex data produced by the microarray (26) and determining the responsible genes. The present study used bioinformatics methods to analyze the functions and pathways of the differentially expressed miRNAs and mRNAs in ESCC, further clarified their biological significance, and defined the key miRNAs and possible target mRNAs affecting the formation of ESCC.

In the present study, a total of 17 aberrantly expressed miRNAs were identified in the ESCC samples, compared to adjacent normal tissues. As the expression of miRNA is known to be tissue- and tumor-specific (27), using the appropriate tumor subset with the corresponding control subset is important to reduce the potential complexities associated with analyzing heterogeneous tumor tissues. The present study aimed to investigate miRNA-mRNA regulation in ESCCs, one of the largest gene expression microarray datasets was used to identify the miRNA targets. Following assessment of the GSE53625 microarray series, 1,670 differently expressed probes were found. The negatively correlated mRNAs with previous differential miRNAs were then used as the base for further investigation of the role of the miRNAs in ESCC.

GO is widely recognized as a premier tool for the organization and functional annotation of molecular aspects (28). By using the criteria of P<0.05, significant GO terms, and the genes involved in them, were obtained. GO terms associated with transcription regulation response are important in ESCC through miRNAs, and this is correlated with the predominant biological function of the miRNAs in humans. Several upregulated GO terms account for cell motility and migration, Matsushima *et al* reported that miRNA-205 modulated ESCC invasion and migration via regulating zinc finger E-box binding homeobox 2 (29). In addition, the cell proliferation term was also observed in this group, revealing increased growth ability in ESCC. By contrast, GO terms in the dowregulated group belonged to the negative behavior of the cell proliferation. Transcriptional regulation is the major function of miRNAs (30), and significant changes in this term observed in the present study further confirmed the results of the present study. Furthermore, previous reports have investigated the role of miRNA in regulating ESCC cell death and revealed promising results (31–33). For example, Wang *et al* (31) demonstrated that miR-22 induces ESCC cell sensitivity to irradiation (34). However, other biological processes may also have effects in ESCC tumorigenesis.

Pathway analysis can reveal distinct biological processes and identify the significant pathways that dysregulated mRNAs are involved in, which can provide a comprehensive understanding of the interactions of genes, their functions and the association between up- and down-stream genes, and can identify genes, which may be regulated by miRNAs. The appearance of the pathways in focal adhesion, gap junctions and cancer pathways confirm their concordance with GO terms and their critical role in ESCC. Focal adhesion has been found to be involved in esophageal cancer migration and invasion (35), however, its molecular mechanism remains to be fully elucidated, and miRNA regulation may be involved. A previous study revealed that cytokines are also involved in the esophageal cancer process, particularly via the mitogen-activated protein kinase (MAPK) pathway (36). LTBP-2, a type of extracellular matrix (ECM) protein, decreases the colony-forming abilities of ESCC and induces tumor suppression (37). The role of miRNAs in ESCC remains to be fully elucidated, and less is understood regarding the associated signaling pathway information regulated by miRNAs. The present study suggested that other, seemingly irrelevant, pathways are controlled by miRNAs and have their functions in ESCC, which requires further investigation. In the present study, the results of the pathway analysis on important roles and functions of miRNAs were similar to those of the GO analysis.

In the present study, the investigation of genes involved in significant GO terms and pathways revealed 164 genes in common that may be regulated by miRNAs in ESCC. miRNA-181a functions as an oncomir in gastric cancer (38), however its role in ESCC remains to be fully elucidated. miRNA-202 is a novel tumor suppressor and is a potential tumor suppressive miRNA involved in the carcinogenesis of human hepatocellular carcinoma (39). It has been demonstrated that miRNA-155 acts as an oncogene by targeting TP53INP1 in ESCC (40). FNDC3B has also been identified in an oncogenomic screen for amplified oncogenes, and over-expression of FNDC3B induces epithelial-to-mesenchymal transition and activates several cancer pathways (41). BNC2 has been identified as a tumor suppressor in esophageal cancer, based on single nucleotide polymorphism microarrays, and transfection and stable expression of BNC2 causes growth arrest of esophageal cancer cells (42). MBD2 is a member of the MBD protein family, the expression of which is reduced in esophageal cancer (43). MBD2 binds to methylated promoter CpG islands and acts as a methylation-dependent transcriptional repressor (44). It has been found to be a target gene of miRNA-224 and miRNA-221^*^ (45). Although their functions have received less investigation, several miRNAs may regulate ESCC. In addition, the differential expression of these miRNAs associated with other clinical characteristics, including smoking and TNM stage, indicated their important role in ESCC. Based on these data, further investigation of the expression and target functions of the identified miRNAs is required, in more samples. In addition, the regulation of the identified miRNAs and pathway functions require investigation, which may assist in improving the clinical diagnosis and treatment of patients with ESCC.

In conclusion, the results of the present study indicated that, by correlating the mRNA and miRNA expression data from two platforms, putative miRNA-mRNA interactions in ESCC were identified. GO and pathway analysis identified pathways controlling the MAPK and peroxisome proliferator-activated receptor signaling pathways, as well as focal adhesion and ECM-receptor interaction pathways. Network analysis also revealed important miRNAs and mRNAs, including miRNA-181a, miRNA-202, miRNA-155, FNDC3B, BNC2 and MBD2, which may be involved in ESCC. Based on the integrated analysis of transcriptome features, these results may provide an important contribution to future investigations aimed at characterizing the role of specific miRNAs in the pathogenesis of ESCC, and contribute to improving diagnosis and treatment.

## Figures and Tables

**Table I:** Clinicopathological characteristics of patients with esophageal squamous cell carcinoma.

Characteristic	GSE13937 n (n=44)	GSE53625 n (n=179)
Alcohol consumption	33	101
Smoking status	33	105
TNM stage		
I	7	12
II	18	86
III	6	81
IV	8	0
NA	5	0

NA, not available. TNM, tumor-node-metastasis.

**Table II:** Collection of dysregulated miRNAs, detected using microarray analysis, in ESCC.

A, Upregulated in ESCC			
miRNA	P-value	FDR	Fold change
hsa-miR-21	1.29×10^−10^	4.01×10^−7^	2.59
hsa-miR-146b-5p	7.03×10^−8^	4.13×10^−5^	1.91
hsa-miR-155	6.16×10^−7^	2.30×10^−4^	1.88
hsa-miR-223	2.51×10^−6^	8.35×10^−4^	2.38
hsa-miR-7	5.66×10^−6^	1.35×10^−3^	1.59
hsa-miR-181b	1.48×10^−5^	2.59×10^−3^	1.47
hsa-miR-224	7.28×10^−5^	7.58×10^−3^	1.82
hsa-miR-181a	1.61×10^−4^	1.22×10^−2^	1.45
hsa-miR-146a	5.10×10^−4^	3.09×10^−2^	1.45

B, Downregulated in ESCC			
miRNA	P-value	FDR	Fold change
hsa-miR-375	6.22×10^−7^	2.30×10^−4^	0.38
hsa-miR-26a-1^*^	8.21×10^−5^	8.08×10^−3^	0.57
hsa-miR-378	9.23×10^−5^	8.79×10^−3^	0.56
hsa-miR-202	1.19×10^−4^	1.05×10^−2^	0.32
hsa-miR-100	2.32×10^−4^	1.58×10^−2^	0.59
hsa-miR-145	5.28×10^−4^	3.14×10^−2^	0.59
hsa-miR-143	7.85×10^−4^	3.90×10^−2^	0.59
hsa-miR-1	8.42×10^−4^	4.07×10^−2^	0.46

ESCC, esophageal squamous cell carcinoma; FDR, false discovery rate; miR, microRNA.

**Table III:** Most markedly dysregulated genes, sorted by FDR, in ESCC tissue compared with normal tissue.

A, Upregulated in ESCC			
mRNA	P-value	FDR	Fold change
COL1A1	7.07×10^−154^	1.27×10^−149^	13.06
COL10A1	1.35×10^−147^	1.61×10^−143^	38.33
MMP1	8.01×10^−134^	4.41×10^−130^	30.41
POSTN	3.21×10^−132^	1.35×10^−128^	16.70
SPP1	6.29×10^−127^	2.05×10^−123^	31.89
AURKA	1.52×10^−125^	4.18×10^−122^	5.36
FSCN1	1.84×10^−122^	4.26×10^−119^	5.17
ADAMTS12	5.51×10^−122^	1.19×10^−118^	17.63
LAMC2	2.96×10^−121^	6.06×10^−118^	11.52
MFAP2	1.66×10^−120^	3.12×10^−117^	12.31

B, Downregulated in ESCC			
mRNA	P−value	FDR	Fold change
EMP1	6.05×10^−148^	8.66×10^−144^	0.08
GCOM1	4.37×10^−147^	4.47×10^−143^	0.05
PPP1R3C	2.21×10^−144^	1.58×10^−140^	0.06
ELN	3.60×10^−142^	2.35×10^−138^	0.13
MAL	2.08×10^−132^	9.30×10^−129^	0.04
MGLL	1.77×10^−128^	6.66×10^−125^	0.18
CRISP2	3.21×10^−127^	1.15×10^−123^	0.01
SH3BGRL2	6.31×10^−127^	2.05×10^−123^	0.07
SASH1	1.55×10^−126^	4.62×10^−123^	0.11
CAB39L	1.07×10^−125^	3.07×10^−122^	0.16

ESCC, esophageal squamous cell carcinoma; FDR, false discovery rate; miR, microRNA.

**Table IV:** Gene Ontology terms significantly upregulated by microRNAs.

Gene Ontology term	P−value	Fold enrichment
Collagen fibril organization	1.88×10^−9^	56.30
Extracellular matrix organization	2.50×10^−7^	17.94
Extracellular structure organization	5.17×10^−6^	11.45
Collagen metabolic process	2.16×10^−4^	33.32
Multicellular organismal macromolecule metabolic process	2.93×10^−4^	30.10
Multicellular organismal metabolic process	4.98×10^−4^	25.22
Cell motion	3.65×10^−3^	3.93
Cell migration	6.01×10^−3^	5.07
Cell adhesion	8.64×10^−3^	3.00
Biological adhesion	8.71×10^−3^	3.00
Cell motility	9.31×10^−3^	4.56
Localization of cell	9.31×10^−3^	4.56
Cell proliferation	9.81×10^−3^	3.74
Sensory organ development	1.57×10^−2^	5.09
Collagen biosynthetic process	2.09×10^−2^	93.30
Fibril organization	2.91×10^−2^	66.64
Regeneration	3.42×10^−2^	10.14
Regulation of cytokine biosynthetic process	3.88×10^−2^	9.46
Response to reactive oxygen species	3.98×10^−2^	9.33

**Table V:** Gene Ontology terms significantly downregulated by microRNAs.

Gene Ontology term	P−value	Fold enrichment
Phosphate metabolic process	7.05×10^−4^	2.78
Phosphorus metabolic process	7.05×10^−7^	2.78
Enzyme linked receptor protein signaling pathway	2.62×10^−3^	4.22
Protein amino acid phosphorylation	3.29×10^−3^	2.97
Regulation of cell development	5.26×10^−3^	5.28
Response to endogenous stimulus	6.57×10^−3^	3.56
Regulation of epithelial cell proliferation	6.88×10^−3^	10.16
Cell fate commitment	7.05×10^−3^	6.49
Transmembrane receptor protein tyrosine kinase signaling pathway	7.60×10^−3^	4.83
Cell morphogenesis involved in differentiation	1.08×10^−2^	4.44
Cell projection morphogenesis	1.09×10^−2^	4.42
Phosphorylation	1.14×10^−2^	2.48
Cell part morphogenesis	1.30×10^−2^	4.23
Cell morphogenesis	1.31×10^−2^	3.55
Response to hormone stimulus	1.51×10^−2^	3.44
Cell projection organization	1.53×10^−2^	3.43
Response to organic substance	1.64×10^−2^	2.50
Cellular component morphogenesis	2.13×10^−2^	3.18
Peptidyl−tyrosine phosphorylation	2.62×10^−2^	11.76
Regulation of system process	2.70×10^−2^	3.50
Peptidyl−tyrosine modification	2.83×10^−2^	11.27
Positive regulation of programmed cell death	3.09×10^−2^	2.92
Positive regulation of cell death	3.15×10^−2^	2.90
Cell adhesion	3.69×10^−2^	2.32
Biological adhesion	3.71×10^−2^	2.32
Regulation of synaptic transmission	3.84×10^−2^	5.31
Negative regulation of transcription, DNA−dependent	4.52×10^−2^	3.04
Cell−cell signaling	4.53×10^−2^	2.40
Regulation of transmission of nerve impulse	4.66×10^−2^	4.91
Negative regulation of RNA metabolic process	4.80×10^−2^	2.99
